# Social Perception Deficit as a Factor of Vulnerability to Psychosis: A Brief Proposal for a Definition

**DOI:** 10.3389/fpsyg.2022.805795

**Published:** 2022-05-11

**Authors:** Álvaro Cavieres, Pablo López-Silva

**Affiliations:** ^1^Escuela de Medicina, Universidad de Valparaíso, Valparaíso, Chile; ^2^Escuela de Psicología, Universidad de Valparaiso, Valparaíso, Chile

**Keywords:** social cognition, social perception, social affordances, psychosis, schizophrenia

## Abstract

Disturbances in social cognition are a core feature of schizophrenia. While most research in the field has focused on emotion perception, social knowledge, theory of mind, and attribution styles, the domain of social perception has received little specific attention. In this paper, we suggest that this issue can be explained by the lack of a precise and unitary definition of the concept, this leads to the existence of different competing uses of the concept and their conflation with other domains of social cognition. Relying on resources coming from the ecological approach to psychology and the phenomenological tradition in psychiatry, we propose that the concept of Social Perception should be used to refer to low-level pre-reflective processes underlying the awareness of interpersonal interactions *with* and *between* others. Clinical data suggests that people with schizophrenia have problems perceiving social situations as opportunities for social engagement, so, in order to fulfil this explanatory need, we propose that the term should be used to capture this important—yet neglected—domain of social cognition. We conclude with the discussion of some future directions for research derived from our proposal.

## The Social Dimension of Schizophrenia

Schizophrenia is linked to a number of social difficulties, among them, reduced capacity for close relationships (Budziszewska et al., 2020), decrease in obtaining and keeping jobs (Hakkaart-van Roijen et al., 2015), decreased engagement in social activities (Bellack et al., 2007), and, in general, a less adequate social functioning (Gorostiaga et al., 2017). Although there is an important degree of causal overlapping with a general deficit in individual cognitive performance, most of these problems have been attributed to poor functioning in social cognition (Schmidt et al., 2011; Halverson et al., 2019). As a theoretical construct, social cognition has been defined as the ability to perceive, interpret, and process social information in real-world settings, or the capacity to be able to construct representations of the relationship between oneself and others and flexibly use these representations to guide social behavior (Green et al., 2008). Disturbed social cognition does not only limit the possibilities of an adequate psychosocial functioning in schizophrenia, but it also can lead to (a) distorted interpretations of the emotions and intentions of others in non-psychotic populations, and (b) false attribution of these emotions and intentions in neutral social contexts, predisposing genetically vulnerable subjects to develop psychotic symptoms (Mier and Kirch, 2017).

Over the last years, impairments in social cognition have been recognized as a core feature of schizophrenia (Green et al., 2015; Javed and Charles, 2018; Montag et al., 2020; D’Arma et al., 2021). Certainly, there are many processes involved in perceiving, interpreting, and generating responses to others intentions, dispositions, and behaviors; in this context, most described evidence point toward impairments in emotion perception, social perception, social knowledge, theory of mind, and attributional style (Ochsner, 2008; Haut et al., 2020; Montag et al., 2020). Broadly speaking, emotion perception has been regarded as the ability to identify the emotions of others accurately; social knowledge refers to the ability to perceive, understand, and appraise implicit and explicit social roles, rules, and context; theory of mind is conceptualized as the ability to interpret someone’s speech or bodily actions in terms of their intentions; and, finally, the concept of attributional style refers to the usual mode of explaining events as a consequence of internal (personal), external (another person), or situational factors (Green et al., 2008; Savla et al., 2013; Montag et al., 2020). From an experimental point of view, research has shown that people with schizophrenia exhibit impaired recognition of emotions conveyed through facial expressions and verbal communication (Savla et al., 2013; Green et al., 2015). They also present problems representing affective mental states, but not more than straightforward cognitive mental states (Savla et al., 2013).

However, it is important to note that little to no research has been dedicated to the understanding of specific problems in social perception. In this paper, we first argue that this shortage in specific research might be explained as a consequence of the lack of a precise and unitary definition of the concept, thus leading to the existence of different competing uses of the notion and their conflation with other domains of social cognition which depend heavily on cognitive processes by which the subject interprets and understands the surrounding environment. Instead, we define social perception as one of the most fundamental functions of being in the world with others. On our view, altered social cognition would be a fundamental feature of schizophrenia as it has been described and linked to the origin of psychotic and deficit symptoms within the classic and contemporary phenomenological tradition in psychopathology (Kraepelin, 1904; Jaspers, 1948; Minkowski, 1966; Blankenburg, 1971; Fuchs, 2015a; Van Duppen, 2017; Parnas et al., 2021, among many others). In our view, psychotic symptoms such as delusions and hallucinations would not only imply disturbed ideas, thoughts, or perceptions. They would also include the impossibility of modifying or relativizing those mental states by sharing them in a common space with others. At the same, negative symptoms would be more than a deficit of expression or motivational drive; they would also be a failure to perceive the opportunity to interact and generate a shared lived reality with others. From a therapeutic point of view, incorporating the notion of a failure of social perception would broaden the current focus of treatment from improving the patient’s cognitive abilities to better understand the world in front of them, to improving their abilities to be in the world with others.

By relying on resources coming from the ecological approach to psychology—especially on the concept of *affordance*—and the phenomenological tradition in psychiatry, we propose that the concept of *Social Perception* should be used to refer to low-level pre-reflective processes underlying the awareness of interpersonal interactions *with* and *between* others. Although direct empirical evidence is lacking, we believe that people with schizophrenia have problems perceiving social situations as opportunities for social engagement. In other words, subjects have problems perceiving social situations as social situations. For this reason, we propose that the term social perception might be useful to capture this important yet neglected domain of social cognition that appears to be altered in schizophrenia (Cavieres et al., 2020). Finally, we conclude by discussing some future directions for research derived from our brief and explorative proposal.

## The Many Faces of Social Cognition

Definitions of *Social Cognition* found in the expert literature tend to differ in scope and the conceptual background underlying their main tenets. More importantly, these notions do not only differ in the way the term is defined or in the emphasis put on the social or individual aspects of social interactions; they also differ in the way in which the concept might—or might not—interact with other domains of social cognition. For example, Allison et al. (2000, p. 267) try to highlight the ability to rapidly perceive the intentions of others. The authors suggest that “social perception is the initial stage of evaluating intentions and psychological dispositions of others by analysis of gaze direction, body movement, and other types of biological motion.” In this view, social perception is—mostly—an evaluative process aiming at translating behavioral clues into relevant information to guide adequate responses in social settings. Contrasting and emphasizing the importance of social context in human interactions, McCleery et al. (2014, p. 54) claim that social perception: “refers to identifying and utilizing social cues to make judgments about social roles, rules, relationships, context, or the characteristics (e.g., trustworthiness) of others.” In this view, social perception would have a more active nature as it includes the process of identifying environmental cues. However, it is not clear the way that this process of identification is guided. McCleery et al. (2014, p. 54) also claim that social perception: “includes social knowledge, which refers to one’s knowledge of social roles, norms, and schemas surrounding social situations and interactions.” As a consequence, it is difficult to identify the specific conceptual differences between the notions of social knowledge and social perception. By contrast, Jacob and Jeannerod (2005) offer a more restrictive view of social perception and claim that only cues of actions and intentions directed toward conspecifics (intentions to affect the other’s behavior) should be included within the domain of social perception.

In the context of the MATRICS initiative, Green and Leitman (2008) define social perception as one’s ability to identify social roles and context including relationship perception, which refers to the perception of the nature of relationships between people. In the authors’ view, social knowledge would refer to the awareness of the roles, rules, and goals that characterize specific social situations and guide social interactions. Pinkham (2014, p. 14), on the other hand, ignores this difference and conceptualizes social perception as the decoding and interpretation of social clues in others, including “the ability to integrate contextual information and social knowledge into judgments of others’ behaviors.” Later on, Green et al. (2015) would revise these definitions, employing the term perception of social clues to refer to the ability to perceive the social information in others’ faces, voices, and body movements, including gait, posture, and gestures. Apparently excluding context, the concept of social-clues perception is divided into (a) facial perception refers to the decoding of affective information from others’ facial expressions, and (b) vocal perception, defined as the recognition and discriminating of acoustic properties of speech and the respective affective information they convey.

A brief literature review reveals the existence of a number of competing definitions of the notion of social perception, and with this, the existence of different alternatives for the way in which social perception might relate to other domains of social cognition (especially social knowledge). A more problematic issue seems to underlie this conceptual debate, namely, the contradictory use of the two basic concepts of the target debate, the concept of “social,” and the concept of “perception.” Within the debate, the concept of “social” is commonly used as a synonym for interpersonal, namely, as the source of information and the context in which this information is relevant. At the same time, perception seems to be used to refer to the initial impression caused by sensory stimuli or the set of cognitive operations originating from these stimuli. Contrasting with the many faces of the term “social perception,” notions such as “social knowledge,” “theory of mind,” “emotion perception and processing,” and “attributional style” seem to be both better-defined and researched within the domain of social cognition. Here, it is important to note that none of the aforementioned domains of social cognition specifically reflect, perhaps, one of the most relevant dimensions when trying to understand social cognition-related phenomena, namely, the capacity to *perceive a social situation as social*, that is, as an instance of interaction with and among others. In the next section, we will explore our proposal by looking closer at the way in which humans perceive the social world.

## Experiencing the Social World: How Do We Socially Perceive?

For human beings, the most prominent features in their environment are other humans. Although the humans we perceive in the environment might share some physical properties with the rest of the material world, we perceive them as different from mere material objects. We are aware of other humans as having emotions and intentions, namely, as having a mental life. However, the most striking difference between humans and other things we perceive is that other humans can meaningfully interact with us and other humans in the environment. In fact, perceiving the emotions and intentions of others helps us to understand these interactions so we can adequately navigate the social world. In a certain sense, situations become social when we become aware of these actual or potential interactions. The world is experienced as a social world as long as it offers opportunities for social engagement. According to Heider (2005), the critical differences between people and inanimate things are that as: (i) persons have phenomenally private experiences such as perceptions, imaginations, thoughts, feelings, and the like, which enable them to act as “centers of action” on the basis of their internal reasoning,^1^ and additionally, (ii) people can establish a peculiar functional closeness and interaction in social encounters if they interact with each other.

Humans can rapidly perceive the emotions experienced by others (emotional perception; Jang and Elfenbein, 2015) and use them, together with other sources of information, to grasp their internal reasoning (Castelli, 2015). Some suggest that this process can take the form of a sequence where perceptual inputs from sensory stimuli are used to generate inferential interpretations of others’ minds (Goldman, 2006; Pérez-Osorio et al., 2021). Others suggest that we can also *perceive* directly (rather than *inferring*) other people’s intentions (Dokic, 2010; Kiverstein, 2015; Gallagher, 2017). Arguably, this is to say that we can perceive emotions and intentions in others, but such stimuli become truly social when they are used for processing cues of actions and intentions of a person directed to affect others’ or our own behaviors.

Dokic (2010) describes two models of joint action with other people. In the first, joint action depends on mind reading. In the second, joint action involves the participants’ non-mentalizing perceptions of social affordances. Adapting Gibson’s definition of the term (see Gibson, 1966, 1977, 1986), we shall understand the term social affordances as the combination of a subject’s features and possibilities for action that people in the environment offer to her and someone else. In this context, affordances can be either ego-centric when the agent is the perceiver or allo-centric when it concerns another agent. The latter type does not require mind reading abilities; all that is needed is the ability to represent other people and their bodily movements. Importantly, the perception of allo-centric affordances for others’ actual or potential actions could reveal previously unperceived ego-centric affordances (and viceversa). This, in turn, would allow the emergence of interpersonal affordances as opportunities for joint actions integrating both ego and allo-centric aspects (Richardson et al., 2007).

This proposal seems to be compatible with the neural correlates of social information processing, comprising two distinct systems: the “mirror neuron system” (MNS) and the “mentalizing” system (MENT). Both systems are activated during interaction or communication with other human beings in social encounters (Bickart et al., 2014; Vogeley, 2017; Porcelli et al., 2019). However, although their precise functional roles are still unclear, it has been proposed that the MNS serves in early stages of social information processing related to the “detection” of spatial or bodily signals, whereas the MENT is recruited during later stages of social information processing related to the “evaluation” of emotional and psychological states of others (Vogeley, 2017; Geiger et al., 2019). While decreased connectivity has been observed both within the MNS and the MENT in subjects with schizophrenia compared to controls, only dysconnectivity of the MNS was related to symptom severity as assessed by the PANSS general and total scale. This could suggest differential patterns contributing to interpersonal difficulties (Schilbach et al., 2016). It has been argued that mirror neurons are linked to the production of information about the observer’s *own* action opportunities, rather than a representation of the other agent’s intention. In Dokic’s terminology, mirror neurons underlie *ego-centric* perceptual representations of what the observer can do in a given situation generated from the perception of others’ actions.

The aforementioned ideas can be connected with the way in which the concept of “intersubjectivity” has been explored within the phenomenological tradition. Fuchs (2015a) claims that as: “Intersubjectivity in its full sense is thus based on the ability to oscillate between an ego-centric, embodied perspective on the one hand, and an allo-centric or decentered perspective on the other, without thereby losing one’s bodily center of self-awareness” (p. 179). Within this perspective, intersubjectivity implies a continuous “co-construction of meaning through mutual interaction and perspective taking” (p. 179). Observing others and their actions immediately opens the possibility of perceiving joint actions. This specific ability to anticipate actual or potential interactions is what we propose to call social perception, separating the term from the other domains of social cognition mentioned in section 1. According to Bickart et al. (2014), both the MNS and the MENT systems interact with other three, partially distinct, brain networks anchored in the amygdala; the network involved in the detection and processing of social stimuli; the social affiliation network and the social aversion network. Detection of social stimuli is rapid and automatic and directs attention to socially salient stimuli with subsequent activation of the other amygdala-centered circuits promoting or inhibiting pro-social behavior as well as the higher-order cognitive processes of the MENT and MNS systems (Porcelli et al., 2019). In sum, information about what others are doing is processed in our brains differently and in parallel to information about what they are feeling. Although this might sound like an oversimplification of a still insufficiently understood process, the brain seems to have a dynamic and hierarchical system of circuitries involved in simpler forms of more automated processing, like the immediate detection of socially relevant stimuli in amygdala-centered circuits, and a partially overlapping circuitry involved in the further interpretation of others actions, intentions, and emotions requiring the activation of the MENT and MNS systems.

## How Social Cognition Goes Wrong in Schizophrenia?

The distinction between general cognitive deficits and social cognitive functioning brought attention to the difficulties that people with schizophrenia have in recognizing other people’s expressions and their emotional states and intentions. However, the lack of a more precise definition of social perception, including its conflation with other domains of social cognition, has resulted in a shortage in research on the specific problems in perceiving and understanding social interactions. Savla et al. (2013) reported the results of a meta-analysis of deficits in different domains of social cognition compared to controls, finding large effects for social perception, theory of mind, emotion perception, and emotion processing. Using a different classification, Green et al. (2015) found evidence of impairments in the perception of social cues (face and prosody perception), mentalizing and emotion regulation with emotion experience, and motor resonance (activation of the MNS) and affect sharing, largely intact in schizophrenia. Lee et al. (2013) reported that people with schizophrenia can use contextual social information provided to help them identify ambiguous facial expressions. Nikolaides et al. (2016) investigated gaze behavior in schizophrenia in relation to social interactions and its impact on social and role functioning. When observing social interaction scenes, subjects showed a shorter scan path length, fewer fixations, and a shorter mean distance between fixations. Furthermore, they exhibited fewer and shorter fixations on faces, but neither on the socially informative bodies nor in the background, suggesting a cue-specific abnormality.

Using a similar task, Sergi and Green (2003) and Sergi et al. (2006) suggested that social perception in schizophrenia is related to alterations in very early aspects of visual processing, although their results were heavily influenced by the educational status of the experimental subjects. Kitoko et al. (2020) report the results of a study using an integrated social perception and knowledge task on people with schizophrenia. Subjects had reduced performance in interpretation and awareness of social conventions. However, these deficits were not correlated with the severity of clinical symptoms, and individual profiles analyses showed a marked heterogeneity among subjects on their abilities. Karpouzian et al. (2016) used a facial affect perception task and video scenes of a single person displaying different facial expressions, voice intonations, and bodily gestures to evaluate social perception in a group of individuals with schizophrenia; they concluded that high, but not low functioning individuals have preserved social perception. In our own research, we asked people with schizophrenia to make sense of a scene depicting an ambiguous social situation with faces, thoughts, and facts about the scene hidden from view (Cavieres et al., 2020). Participants were required to select a limited number of these items before providing an answer. People with schizophrenia—as well as controls—had a strong preference for knowing the thoughts of the characters and their interpretations did not differ from the control group. The results of this study suggest that, despite difficulties perceiving clues about the mental state of others, people with schizophrenia use this information when trying to understand social situations.

From a neurobiological perspective, Ebisch et al. (2017) examined the relationship between task-evoked neural activity during observation of social interaction and the functional organization of self-related brain networks during the resting state. Task-evoked activity in the dorsal posterior cingulate cortex during social perception co-varied with dorsal posterior cingulate cortex-ventromedial prefrontal cortex functional connectivity during resting state in controls not in people with schizophrenia. Using a different paradigm, they also reported the involvement of the dorsolateral prefrontal cortex and superior temporal sulcus in impaired social perception in schizophrenia. What these results show is that people with schizophrenia present problems in different and separate dimensions of social cognition, not only in perceiving the emotions and mental states of others and constructing hypotheses about their intentions but also in perceiving potential or actual interactions. This specific alteration might hinder the possibility of experiencing a social world where interactions with others can be had and shared.

The aforementioned idea seems to be present in some of the earliest descriptions of schizophrenia (e.g., in the descriptions offered by Bleuler and Minkowski). Later, psychopathological descriptions of the condition seem to have focused on psychotic symptoms and failures in the construction of the self. Nonetheless, disturbances of basic self-awareness and attunement to the social world seen in schizophrenia also impair the subject’s ability to interact with others, not because they lack explicit knowledge, inferential or ToM abilities, but rather, because they seem to lack an implicit understanding of the “rules of the social game”; a sense of proportion for what is appropriate, likely and relevant in the social context (Fuchs, 2015a). From a phenomenological perspective, disturbances of pre-reflective self-awareness and embodiment—including a weakening of the basic sense of self and a disruption of implicit bodily functioning in the dimensions of both perception and action (Sass and Parnas, 2003, 2007)—would lead to a fundamental disturbance of the pre-reflective, embodied, and practical immersion of the self in the (social) world (Van Duppen, 2017).

## How Can Failures in Social Perception Lead to Psychotic Symptoms?

Early authors within the phenomenological tradition in psychiatry such as Bleuler, Kretschmer, and Minkowski considered interpersonal difficulties as a fundamental psychotic vulnerability, a hypothesis that has also been held by other authors in more recent times (Fuchs, 2015a,b; Pienkos, 2015; Henriksen and Nilsson, 2017; Van Duppen, 2017; Van Duppen and Feyaerts, 2020). All these authors seem to agree that, beyond difficulties in other areas of social cognition, schizophrenia would be characterized by a basic impairment in social perception in the sense in which we have just defined it in the previous sections. People with schizophrenia can recognize and identify other human beings and their actions but experience difficulties perceiving the interpersonal affordances of their presence. This can be interpreted as an inability to generate an adequate intersubjective space to share and construct meanings about everyday life experiences, including the use of pragmatic language. This leaves subjects vulnerable to the emergence of idiosyncratic and self-referential interpretations that might produce the core of psychotic experiences, especially delusions. Moreover, without an effective form of intersubjective communication, subjects also lose the possibility of developing metacognitive control over their own ideas. Thus, instead of common, shared meanings of everyday experiences, people with schizophrenia construct their peculiar interpretations in ways that are incomprehensible to others and which, escaping all metacognitive control, are held with apodictic certainty, foregoing verification or relativization in an intersubjective context.

Stanghellini (2001) employs the concept of *common sense* to refer to everything that members of a given society consider as obvious. It involves both practical knowledge about social situations and a basic intuitive attunement (social perception) with the social world aimed at understanding these situations. Whereas the concept of “social knowledge” refers to the background of constructs useful for organizing the everyday experiences, the concept of “attunement” reflects the affective-conative capacity to get involved in others’ lives and to catch context-relevant cues to make sense of the others and of situations. According to Stanghellini, people with schizophrenia lose contact with this common sense through three mechanisms: (a) sensory (aberrant perception of self, body, and world), (b) typification disorders (disturbances of social knowledge network or lack of attunement), and (c) Attitudinal disorders (distrust toward common sense). These levels of vulnerability are coherently related to each other, in a way that, when intuitive attunement is disordered, not only do the others appear enigmatic and the social environment becomes uncanny, but also one’s sense of the self and the boundaries between oneself and the others may become blurry. All these disruptions would lead to the onset of psychosis.

Fuchs (2015b) explicitly distinguishes between a faulty development or functioning of ToM module “inside” the subject (which renders other persons’ thoughts, feelings, and actions strange and inaccessible) from an immediate, pre-reflective disturbance in the relationship between self and others in an emergent bi-personal field. When the perspectives of self and others are confused instead of being integrated, the subject may be left susceptible to experience symptoms where the subject’s boundaries are diminished or altered such as thought-broadcasting, thought insertion, and auditory-verbal hallucinations. In this sense, traditional approaches focus on failures of self-integration and source monitoring as explanations for auditory hallucinations. Instead, Bell (2013) highlights “social” features of hallucinations such as possessing identities performing communicative acts and being perceived as interlocutors (McCarthy-Jones et al., 2014). There is also evidence that the neural circuits involved in the neurobiology of hallucinatory voices also perform tasks related to social cognition (Rushworth et al., 2013). Rushworth hypothesizes that auditory-verbal hallucinations arise from the internalization of familiar people and their voices, which are used to make inferences about their behavior in imaginary situations. However, further research is needed in order to support this view in light of updated characterization of auditory hallucinatory phenomena (Woods et al., 2015; Rosen et al., 2016).

In relation to delusions, Fuchs (2015a) has argued that we create meanings about our experiences in a continuous circular process of mutual understanding, negotiation of intentions, alignment of perspectives, and reciprocal correction of perceptions—with others in every interaction and communication. However, if there are failures in the establishment of boundary conditions to these circular processes, then the co-construction of meaning will be disturbed, and mutual understanding will fail. These may include perceptual, cognitive, and affective alterations characteristic of schizophrenia, especially in its early stages. Here, our proposal integrates the specific ability to perceive social situations as shared instances with others (social perception). Delusions may be incompletely understood as merely individual false beliefs, they arise when the basic trust, that could help restore a consensual understanding of a situation and co-constitute a shared, commonsensical reality, is missing. Deluded subjects might be able to perceive (emotion recognition), understand, or even take the perspective of others (theory of mind); what they lack in our view is the independent position from which they could compare and integrate their own and another’s point of view. Thus, among other factors, delusions might result from the failure of co-constituting the world through mutually taking and aligning one’s perspectives.

## Conclusion and Future Directions

Disturbances in social cognition are one of the core features of schizophrenia. Most described socio-cognitive deficits in schizophrenia refer to impairments in emotion perception, social perception, social knowledge, theory of mind, and attributional style (Ochsner, 2008; Montag et al., 2020). We have noted an important shortage in specific research regarding social perception. We have proposed that this issue arises from the lack of a precise and unitary definition of the term, leading up to the existence of competing notions about the scope and the way in which social perception would interact with other dimensions of social cognition. In order to deal with this conceptual gap, we have proposed that the concept of Social Perception should be reserved to refer to low-level pre-reflective processes related to the awareness of interpersonal interactions *with* and *between* others. From this point of view, alterations in social perception might capture the fact that people with schizophrenia have problems perceiving social situations as social or, in other words, perceiving opportunities for social engagement.

It is important to note that our proposal is very explorative. Indeed, more research is needed to define the most appropriate way to study social perception and characterize its alterations in the different stages of schizophrenia including people at high risk of psychosis and its relation to the various symptoms of the condition. From a therapeutic and preventive approach, clarifying the relationship between social perception, intersubjectivity, and psychotic experiences and symptoms in schizophrenia is a critical issue that could potentially lead to new forms of psychosocial interventions or to the improvement of existing programs.

Much of the research cited in this article has been subject of criticism regarding the methodology employed. Teufel et al. (2013) pointed out that simple pictorial representations of other people are “social” only in the restricted sense that they reproduce their physical features but not their spatiotemporal properties. Even watching videos can be considered “non-social,” because the observer knows that he is not seeing real persons and will not attribute mental states to the stimuli or do so qualitatively different from real social interaction. Pönkänen et al. (2011) demonstrated different information-processing mechanisms depending on whether or not the observers believe they are seeing a real person present at the time of viewing. Teufel et al. (2010) believe that another person’s actual or assumed presence increases the tendency of observers to attribute mental states to viewed stimuli, which affects the perceptual processing of specific socially relevant information.

Regardless of the precise sequence in which it occurs, it seems clear that perceiving other people, and their actions, is a distinct process from emotion recognition and mindreading. Consequently, we believe that an ideal research design for this domain should be able to specifically investigate the ability to perceive other subjects as interacting with each other, independently of adequately identifying their intentions or emotional states. Moreover, being able to describe these actual or potential interactions does not necessarily require knowledge of the rules that apply to a specific social situation. For example, in our own unpublished research, patients recognize a group of subjects performing different tasks, following their own intentions in a common situation (opening the fridge, fetching cups, and boiling water for tea) but do not perceive them as performing a common task (preparing to have breakfast together). Normally, we observe others interacting with the world and the objects in it, but most importantly, with other persons and with ourselves. This is what allows us to perceive others as social intentional agents, whose actions and interactions are purposeful in contexts shared with others, and to learn to experience ourselves as intentional agents perceived by others in shared social spaces.

Fuchs (2015b) criticizes current paradigms based on a conception of the subject with schizophrenia as an enclosed individual with a clearly defined brain dysfunction. Instead, he states, most mental disorders imply more or less profound disturbances of intersubjectivity, which means, a restricted capacity to respond to the social environment in a flexible way and to reach a shared understanding through adequate interaction with others (p. 191). Nevertheless, emotion recognition, mind reading, attributional styles, and social knowledge all occur mostly within the subject and so, are commonly studied in contexts with no real interactions. For instance, there is an important line of research and therapy for people diagnosed with schizophrenia based on the importance of the metacognitive aspects defined as an activity carried out by one person in order to know, monitor, and adjust their beliefs, memories, and behaviors (Moritz and Woodward, 2007). However, instead of a cognitive activity concretely located in an isolated mind, metacognition is entwined with intersubjective experience.

Psychosocial rehabilitation programs could benefit from incorporating this view in their design and methodology, favoring safe and shared working environments with other patients and therapists, where it is possible to examine experiences in a non-judgmental way and facilitate the incorporation of interpretations that include others’ points of view, but above all, favoring constructive and real-life oriented social interactions. This could result in the generation of an intersubjective psychic space where it is possible to relativize one’s own experiences and opinions without weakening ego-identity and self-confidence. It could be relevant in this sense to measure the impact of the programs on aspects such as trust in others, satisfaction with the therapy, improvement in self-esteem, and social skills.

We propose to define social perception as the awareness and comprehension of actual and potential interactions with and between others, including the understanding of individual and shared roles and intentions conveyed by expressions, speech, and behavior directed to other subjects and within a social environment. As such, this domain should be assessed independently from the ability to recognize facial expression and of mind reading, ideally in ecologically valid settings that allow re-enactment of emotionally relevant social interactions from a first-person perspective. Appraisals of experiences, including one’s own thoughts, wishes, and feelings as well as the outcome of behavior, are necessarily shaped by the relative meanings assigned to different aspects of that experience. Those meanings that are assigned to an aspect of experience are naturally influenced and molded by how others do or might perceive and think about either those experiences or to how one is interpreting those experiences. Hasson-Ohayon et al. (2020) have reflected on this issue and proposed that while evidence is accumulating about the relationship between metacognition and recovery from schizophrenia, the role of intersubjectivity in the recovery process has been less studied. While we have explored literature on substantial alterations in metacognition, it is unclear to what extent intersubjective experience plays a role in persons who experience more negligible alterations. In addition, while we have advocated intersubjective consideration into the treatment of persons with schizophrenia, this is yet to be fully supported empirically.

## Author Contributions

AC and PL-S contributed to the conception and writing of the manuscript. All authors contributed to the article and approved the submitted version.

## Conflict of Interest

The authors declare that the research was conducted in the absence of any commercial or financial relationships that could be construed as a potential conflict of interest.

## Publisher’s Note

All claims expressed in this article are solely those of the authors and do not necessarily represent those of their affiliated organizations, or those of the publisher, the editors and the reviewers. Any product that may be evaluated in this article, or claim that may be made by its manufacturer, is not guaranteed or endorsed by the publisher.

## Footnotes

^1^It is important to note that Heider’s definition might also apply to animal perception. However, an analysis of this issues goes beyond the focus on our current work.
